# Combination of Treadmill Training and Inosine Enhance Nerve Regeneration and Functional Recovery After Mice Sciatic Nerve Transection

**DOI:** 10.1002/jnr.70080

**Published:** 2025-09-16

**Authors:** Tiago Batos Taboada, Luiza dos Santos Heringer, Camila Linhares Fernandes de Oliveira, Gabriel Valladares da Rosa, Fernanda Marques Pestana, Ricardo Cardoso, Fellipe Soares dos Santos Cardoso, Roberta Ramos Cavalcanti, Bruna dos Santos Ramalho, Ana Maria Blanco Martinez, Fernanda Martins de Almeida

**Affiliations:** ^1^ Neurodegeneration and Repair Laboratory, Graduate Program in Pathological Anatomy, Faculty of Medicine, Federal University of Rio de Janeiro Rio de Janeiro Brazil; ^2^ School of Health Sciences and Wellness Souza Marques College Rio de Janeiro Brazil; ^3^ Institute of Biomedical Sciences, Federal University of Rio de Janeiro Rio de Janeiro Brazil; ^4^ Graduate Program in Rehabilitation Sciences, Faculty of Physical Therapy Federal University of Rio de Janeiro Rio de Janeiro Brazil

**Keywords:** inosine, nerve guidance conduit, nerve regeneration, peripheral nerve injury, treadmill training

## Abstract

Peripheral nerve injuries are a major cause of disability, leading to significant sensorimotor impairment and functional loss. These injuries can result from traumatic or non‐traumatic events, with severe cases posing therapeutic challenges. Neurorrhaphy is the gold standard for treating injuries where the gap between nerve stumps is less than 3 cm, while autografting is used for larger gaps. Despite various therapeutic approaches aimed at enhancing peripheral nerve regeneration, restoring pre‐injury function remains difficult in clinical practice, prompting the exploration of experimental therapies. This study examined the effects of treadmill training and inosine treatment on sciatic nerve regeneration after transection in mice. Male C57/Bl6 mice (8–12 weeks) underwent sciatic nerve transection, with the proximal and distal stumps sutured to a polylactic acid tubular graft, creating a 3 mm gap. The mice were treated with saline or inosine (70 mg/mL) for 1 week, followed by treadmill exercise starting in the second week. The exercise protocol involved treadmill speeds of 6–12 m/min, three times per week for 10 min, continuing for 8 weeks. Functional recovery was assessed weekly using the Sciatic Functional Index, pinprick test, and Von Frey electronic analgesiometer. At the end of the study, electrophysiological tests and morphologic analysis were performed. The results showed that the combination of inosine with treadmill training significantly accelerated functional recovery and nerve regeneration, suggesting that this combined approach may offer a promising alternative for improving recovery outcomes in cases of peripheral nerve injury.

## Graphical abstract

This is a schematic representation of the experimental design and main findings. Mice underwent sciatic nerve transection, which was then repaired using a PLA conduit. The mice were then treated with inosine and/or treadmill training. Combining inosine and treadmill training enhanced axonal regeneration synergistically, improving electrophysiological parameters, increasing neuronal survival and preserving muscle morphology. This ultimately led to superior functional recovery compared to single therapies.

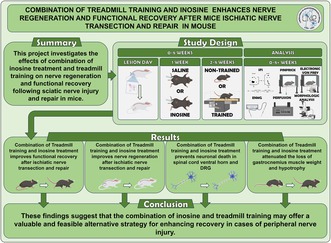

**Summary**

- Peripheral nerve injuries (PNIs) are known to cause severe motor and sensory dysfunction, representing a major clinical challenge with limited functional recovery using conventional approaches.
- Inosine, a purine nucleoside, has been demonstrated to promote axonal sprouting, neuroprotection, and anti‐inflammatory effects, while treadmill training (TT) has been shown to enhance neural plasticity, neurotrophin expression, and motor reinnervation.
- The present study investigated the effects of inosine and TT, either alone or in combination, on sciatic nerve regeneration in mice following transection and repair with a PLA conduit.
- The combined therapy significantly accelerated functional recovery (SFI, pinprick, Von Frey), improved electrophysiological outcomes (CMAP amplitude, latency), enhanced myelination, and increased neuronal survival in the spinal cord and dorsal root ganglia (DRG).
- Furthermore, the combination of these factors led to a reduction in gastrocnemius muscle atrophy and the preservation of muscle fibre structure, thereby supporting the process of functional reinnervation.
- The findings demonstrate that inosine plus TT has synergistic effects, offering a promising multimodal strategy for peripheral nerve repair and functional recovery.

## 1 Introduction

Peripheral nerve injuries (PNIs) represent a significant clinical challenge, frequently resulting in substantial motor and sensory dysfunction and long‐term disability (Modrak et al. 2020; Murphy et al. 2023; Lavorato et al. 2023). Although the peripheral nervous system (PNS) possesses an intrinsic regenerative capacity, complete functional recovery remains rare, particularly in cases involving large axonal gaps or delayed intervention (Lopes et al. 2022; Lee et al. 2022).

Autologous nerve grafting is currently the gold standard for bridging nerve gaps, but it is limited by donor site morbidity, tissue availability, and variable outcomes (Wang et al. 2019; Liu and Duan 2023; Khaled et al. 2023). As a result, nerve guidance conduits (NGCs), especially those made from biodegradable polymers like poly (L‐lactic acid) (PLA), have emerged as viable alternatives. These conduits provide structural scaffolding and a conducive microenvironment for axonal regrowth (Pestana et al. 2018; Kaplan and Levenberg 2022).

Nevertheless, the recovery of full function after nerve repair remains suboptimal. To enhance regeneration, researchers have explored the combination of biomaterials with pharmacological and physical therapies (Goulart and Martinez 2015; Cao et al. 2024). Among molecular candidates, inosine—a purine nucleoside—has shown promise due to its neuroprotective, anti‐inflammatory, and axogenic properties. Inosine enhances axonal sprouting via activation of adenosine A2A receptors, upregulates GAP‐43 expression, and modulates immune responses (Benowitz et al. 1999; Irwin et al. 2006; Cardoso et al. 2019, 2024).

In parallel, physical rehabilitation strategies such as treadmill training (TT) have demonstrated efficacy in promoting functional recovery after PNIs. TT stimulates motoneuron activity, enhances neurotrophin expression (e.g., BDNF, NGF), induces neuroplasticity, and supports reinnervation of target muscles (Sabatier et al. 2008; Gordon 2020; Jaiswal et al. 2020). It has also been shown to regulate epigenetic mechanisms related to axonal regeneration (Davaa et al. 2021).

Despite the established benefits of inosine and TT as standalone therapies, few studies have explored their combined application. Based on their complementary mechanisms—biochemical stimulation of axonal growth and activity‐dependent plasticity—we hypothesized that combining inosine treatment with TT would potentiate nerve regeneration and functional recovery more effectively than either intervention alone. To test this hypothesis, we used a murine model of sciatic nerve transection repaired with a PLA conduit and evaluated functional, electrophysiological, and histological outcomes over an 8‐week period.

## 2 Materials and Methods

### 2.1 Experimental Procedure

To analyze the effects of TT and inosine on sciatic nerve regeneration after transection injury and nerve conduit repair in mice, the experimental design included four groups of animals, each subjected to surgery followed by specific treatments. The Saline group received an intraperitoneal injection of saline solution, while the Inosine group received an intraperitoneal injection of inosine solution. Additionally, animals in the Saline + TT group were treated with saline solution and underwent a TT protocol. Similarly, the Inosine + TT group received an inosine solution and participated in the TT protocol.

### 2.2 Nerve Transection and Repair

Adult male C57/Bl6 mice (8–12 weeks old) were weighed and anesthetized with intraperitoneal ketamine (100 mg/kg) and xylazine (15 mg/kg). Once anesthetized, the right hind limb was shaved, and the sciatic nerve was exposed via a longitudinal incision on the posterior thigh, without muscle incision. Using ophthalmic microscissors, the sciatic nerve was transected 2 mm from the greater sciatic foramen. The proximal and distal nerve stumps were sutured to a 5 mm poly(L‐lactic acid) (PLA) conduit, leaving a 3 mm gap between them, using 10–0 nylon sutures. After tubulization, the muscle was repositioned, and the skin was sutured with 6–0 nylon. Mice were then transferred to recovery cages with a 12‐h light/dark cycle and free access to food and water. The study was approved by the Ethics Committee on Animal Use in Research at the Federal University of Rio de Janeiro (protocol no. 089/22).

### 2.3 Treatment Administration

One hour after sciatic nerve transection and repair with a PLA conduit, animals were randomly assigned to receive daily intraperitoneal injections of either inosine (260 mM—70 mg/kg body weight, Sigma‐Aldrich) or 0.9% saline for seven consecutive days (Kim et al. 2013). Treatment began 1 h post‐surgery and was completed before the first functional assessment or TT session.

### 2.4 Treadmill Training Protocol

The mice underwent a 1‐week treadmill acclimatization before the experiment. TT began on day 7 post‐injury, with mice placed on a motorized treadmill at 6–12 m/min, adjusted for their ability. Sessions lasted 10 min, 3 times weekly, following a modified protocol (Goulart et al. 2014; Massoto et al. 2020). Training continued for 7 weeks until 8 weeks post‐surgery. Saline and inosine groups were placed on a stationary treadmill for the same duration to minimize stress from handling.

### 2.5 Functional Analyses

Functional analyses were conducted preoperatively and weekly for 8 weeks post‐injury. Locomotor performance was assessed using the Sciatic Functional Index (SFI; Inserra et al. 1998). Pain sensitivity was evaluated with a pinprick test, while tactile sensitivity was measured using the Von Frey electronic analgesiometer. The sample size for these analyses was *n* = 6.

#### 2.5.1 Sciatic Functional Index

To analyze the SFI, we used a 45 × 6.5 cm corridor where the animals walked to record their paw prints on white Canson paper. Their hind paws were painted with black water‐based paint, and prints were obtained as the animals walked. Only prints from moderate‐speed walks were selected for analysis. If needed, tests were repeated to get measurable prints (Inserra et al. 1998).

For the SFI calculation, we measured toe spread (TS)—the distance between the first and fifth toes—and print length (PL)—the distance from the third toe to the heel pad. These values were measured for both the experimental (E) and normal (N) sides and calculated using the SFI formula (Inserra et al. 1998).

#### 2.5.2 Pinprick Test

To evaluate cutaneous pain sensitivity, we used the pinprick test. Animals were placed in acrylic boxes with a grid floor made of 1 mm thick, malleable wire, arranged in 5 mm^2^ squares. Mirrors positioned 25 cm below the boxes helped visualize the paw pads. After acclimatization, an entomological pin (0.25 mm diameter, stainless steel) was gently pressed against the plantar surface of the hind paws without piercing the skin. The test score ranged from 0 (complete loss of cutaneous pain sensitivity) to 5 (normal sensitivity) (Ma et al. 2011).

#### 2.5.3 Electronic Von Frey Analgesiometer

To assess mechanical sensitivity, we used the electronic von Frey Analgesiometer (Model EFF 302—Digital Analgesiometer, Insight Instruments), which measures pressure in grams (g) with 0.1 g accuracy. The device was calibrated to record up to 150 g, maintaining accuracy up to 80 g. Pressure was applied using a 0.5 mm disposable polypropylene tip to the mouse's paw through a mesh grid. Mice were placed in the mirrored apparatus described earlier, and pressure was gradually increased until a paw withdrawal response occurred. The recorded value at withdrawal was noted. Three measurements were taken, and the average was calculated.

### 2.6 Electroneuromyography and Nerve Conduction

For axonal regeneration analysis post‐sciatic nerve transection and repair, electroneuromyography was conducted. Mice were anesthetized intraperitoneally with ketamine (100 mg/kg) and xylazine (15 mg/kg), and the sciatic nerve, gastrocnemius muscle, and tendon were re‐exposed. Electrical stimulation was applied to the sciatic nerve using a bipolar hook electrode (cathode 2 mm distal to anode) with the PowerLab 4/35 device (AdInstruments). Needle electrodes recorded compound muscle action potentials (CMAP), with the active electrode in the gastrocnemius muscle, reference on its tendon, and ground under the skin. A 10 V stimulus was applied, and CMAP was analyzed via LabChart 8 software. CMAP amplitude (mV), latency (ms), and nerve conduction velocity (NCV, m/s) were evaluated. NCV was calculated using the time between proximal and distal stimulation sites. The sample size for these analyses was *n* = 4.

### 2.7 Light Microscopy

**FIGURE 1:**
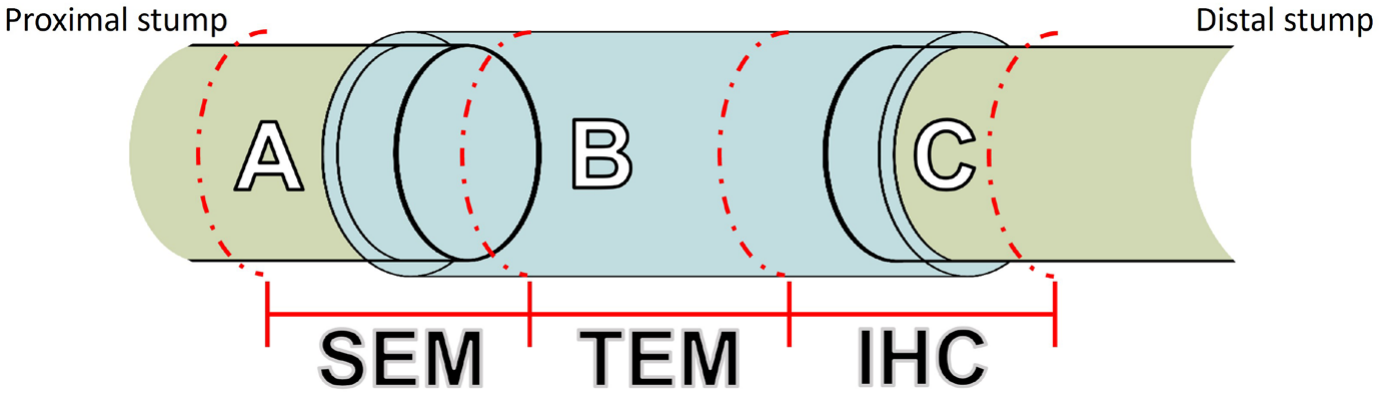
Illustrative diagram of the three segments of the nerve and their respective processing. (A) Proximal nerve segment processed for scanning electron microscopy (SEM). (B) Mid‐regenerated nerve segment processed for transmission electron microscopy (TEM). (C) Distal segment to the lesion processed for immunohistochemical (IHC) analyses.

Eight weeks after injury, animals were anesthetized with ketamine (100 mg/kg) and xylazine (15 mg/kg) and perfused intracardially with 4% paraformaldehyde (PFA) in 0.1 M phosphate buffer or PFA + 2% glutaraldehyde. After perfusion, tissues were collected: right and left gastrocnemius muscles, sciatic nerves (right injured, left control), spinal cord at the L4–L5 level, and dorsal root ganglia (DRG) at L4–L5 bilaterally. The sciatic nerve was divided into three segments (Figure 1) for analysis. Following dissection and post fixation, selected tissues (sciatic nerve segment C, spinal cord, and DRGs) were washed in phosphate buffer and cryoprotected with 10%, 20%, and 30% sucrose solutions. After cryoprotection, tissues were embedded in OCT (Tissue Tek) and sectioned using a cryostat (Leica CM 1850): 10 μm sections for sciatic nerves, 12 μm for DRG, and 20 μm for spinal cord. Sections were mounted on gelatin‐coated slides for immunohistochemistry (IHC) and routine staining.

### 2.8 Transmission Electron Microscopy

For electron microscopy, Segment B of the sciatic nerves was used for electron microscopy. Tissues were washed thrice in 0.1 M CaCO buffer, then postfixed for 2 h in a mix of 1% OsO_4_, 0.8% potassium ferrocyanide, and 50 nM calcium chloride in the same buffer. Samples were rinsed and stained with 1% uranyl acetate. Following three washes in 0.1 M phosphate buffer (pH 7.4), tissues were dehydrated in graded acetone (30%–100%), embedded in epoxy resin, and polymerized at 60°C for 48 h. Post‐polymerization, blocks were trimmed, and 300 nm sections cut with an ultramicrotome (MT‐600‐XL‐RMC Inc.), stained with toluidine blue, and analyzed via light microscopy (Zeiss Axioscope 2 plus). Ultra‐thin sections (60–70 nm) were collected on copper grids, contrasted with 5% uranyl acetate (30 min) and 1% lead citrate (10 min), and examined using a transmission electron microscope (Zeiss 900) at 80 kV with 6000× magnification.

### 2.9 Morphometric Assessment

For morphometric analysis, samples from five areas of transverse semi‐thin sections were taken at 100× magnification. Parameters calculated included fiber area, axon area, myelin area, and G‐ratio. Myelin area was determined by subtracting axon area from fiber area. The G‐ratio was calculated by dividing inner axon diameter by outer fiber diameter, categorized into ranges of 0–0.12, 0.13–0.26, 0.27–0.40, 0.41–0.54, 0.55–0.68, 0.69–0.82, and 0.83–1.0. The mean *G*‐ratio was also calculated. The optimal *G*‐ratio for the sciatic nerve is 0.55–0.68 (Chomiak and Hu 2009). The sample size for these analyses was *n* = 3.

### 2.10 Immunohistochemistry

To analyze neurofilament presence and localization, we used immunohistochemical staining for NF200 (SIGMA, 1:1000) and A2A receptor (Invitrogen, 1:200) for nerve regeneration study. Slides were washed twice in phosphate‐buffered saline (PBS) pH 8.0, blocked with 10% normal goat serum in PBS‐Triton (0.3%) for 1 h at room temperature, then washed and incubated overnight with the primary antibody at 4°C. The next day, slides were washed, incubated with secondary antibody (Alexa 488 and 546, 1:600) for 2 h, washed again, and mounted with Fluoromount (SIGMA‐4680). Analysis was done using a fluorescence microscope (Zeiss Axioscope 2 plus). The sample size for these analyses was *n* = 3.

### 2.11 Motoneurons and Sensory Neurons Quantification

To assess the neuroprotective effect of TT and inosine, motoneurons in the spinal cord's anterior horn (L4 and L5) and sensory neurons in the DRG were quantified. Using 5 transverse sections (20 μm) stained with 0.1% cresyl violet (Nissl), motoneuron nucleoli were quantified from 20× micrographs. A line was drawn perpendicular anterior to the spinal cord's central canal to ensure only motoneurons were counted. Sensory neuron nucleoli in the DRG were quantified using eight 12‐μm thick sections stained with 0.1% cresyl violet (Nissl). Analysis was performed at 20×. The sample size for these analyses was *n* = 3.

### 2.12 Muscle Analysis

To analyze the gastrocnemius muscles' dry weight, tissue samples fixed in 4% paraformaldehyde were weighed after drying with filter paper until constant. The ipsilateral muscle's dry weight was normalized to the contralateral side. For analyzing muscle fiber number and area, tissues fixed in 4% PFA were washed in 0.1 M phosphate buffer and cryoprotected in 10%, 20%, and 30% sucrose in the same buffer, then frozen in OCT (Tissue Tek) and stored at −20°C. 20 μm transverse sections were obtained using a cryostat (Leica CM 1850), and four serial sections were stained with hematoxylin and eosin (HE) after oven‐drying at −57°C for 10 min and OCT removal. Sections were stained in hematoxylin for 3 min, eosin for 2 min, dehydrated in graded ethanol solutions, cleared in xylene, and visualized by light microscopy. The sample size for these analyses was *n* = 3.

### 2.13 Statistical Analysis

For statistical analysis, data were analyzed using GraphPad Prism 9 and presented as mean ± standard error of the mean (SEM). For functional recovery outcomes (SFI, pinprick, and Von Frey tests), two‐way repeated measures ANOVA (treatment × time) was applied to assess treatment and time effects. Tukey's post hoc test was performed only when a significant interaction was detected. For morphological and electrophysiological data involving multiple groups, one‐way ANOVA with Tukey's post hoc test was used for parametric data and the Kruskal–Wallis test for non‐parametric data. A significance threshold of *p* < 0.05 was adopted for all analyses.

## 3 Results

### 3.1 Combination of Treadmill Training and Inosine Treatment Improves Functional Recovery After Sciatic Nerve Transection and Repair

**FIGURE 2:**
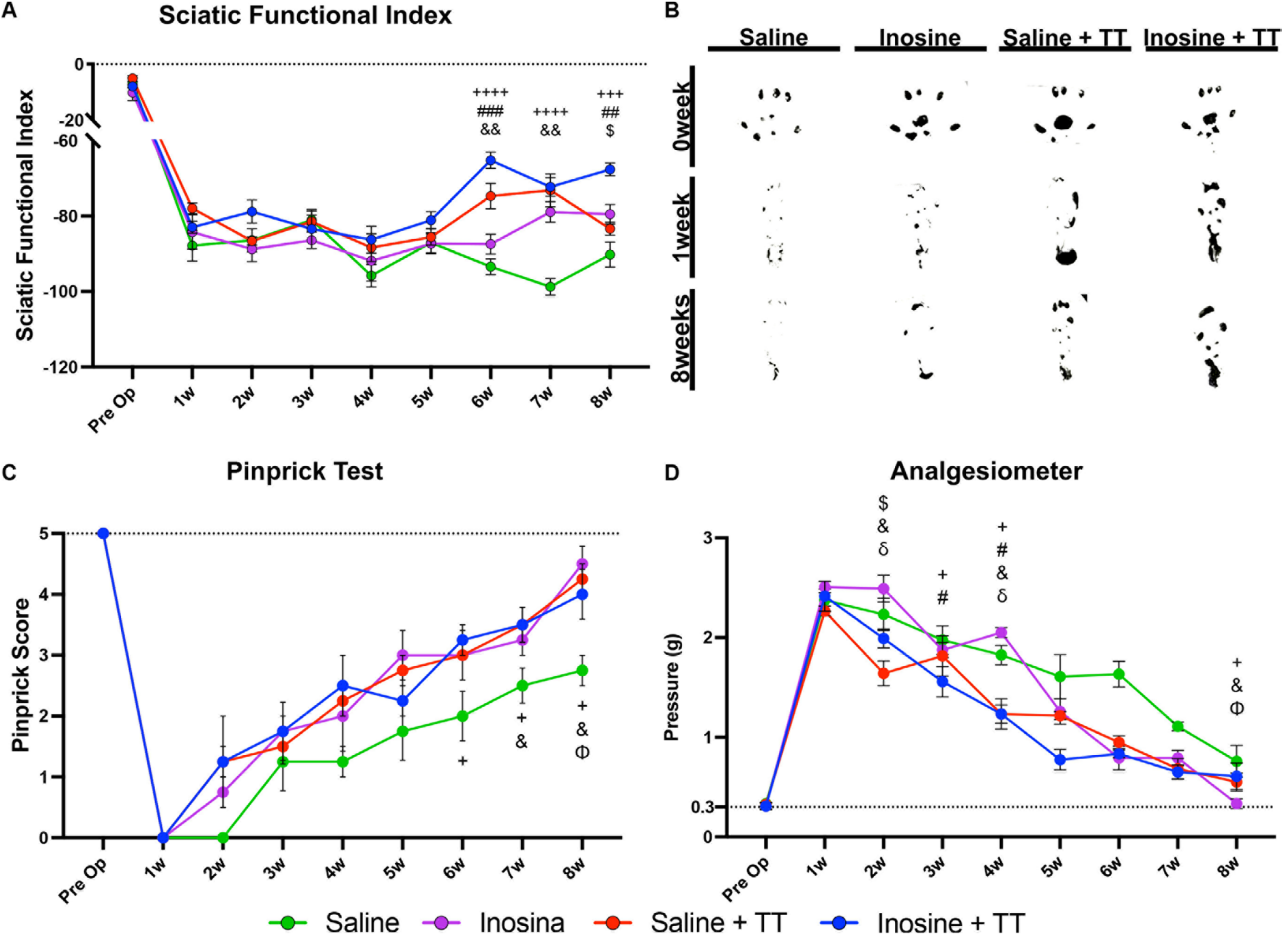
Functional assessment after sciatic nerve injury. (A) Graph showing SFI scores through the weeks. (B) Footprints at the day before lesion, 1 week and 8 weeks after injury. (C) Pinprick scores after the crush injury. (D) Graph showing paw withdrawal threshold in grams on electronic Von Frey analgesiometer. For functional assessment: Saline *n* = 6, inosine *n* = 6, saline + TT *n* = 6 and inosine + TT *n* = 6. Values represent mean ± SEM. (+ for Inosine + TT vs. Saline; # for Inosine + TT versus Inosine; $ for Inosine + TT versus Saline + TT; & for Saline + TT versus Saline; δ for Saline + TT versus Inosine; Φ for Inosine versus Saline for +, #, &, $, Φ and δ, *p* < 0.05; for ## and &&, *p* < 0.005; for +++ and ###, *p* < 0.0005; for ++++, *p* < 0.0001).

To assess locomotor function recovery following sciatic nerve injury, the Sciatic Functional Index was applied (*n* = 6) (Figure 2A,B). At 6 weeks post‐injury, the Inosine + TT group (−65.19 ± 2.16) exhibited significantly better functional scores compared to both the Saline group (−93.42 ± 2.09; *p* < 0.0001) and the Inosine group (−87.43 ± 2.63; *p* < 0.0004). The Saline + TT group (−74.66 ± 3.36) also outperformed the Saline group (*p* = 0.0058). By the seventh week, both Inosine + TT (−72.25 ± 2.45) and Saline + TT (−73.14 ± 4.35) maintained superior performance relative to the Saline group (−98.74 ± 2.17; *p* < 0.0001 and *p* = 0.0078, respectively). At the end of the survival period, the Inosine + TT group (−72.25 ± 3.78) continued to demonstrate superior recovery compared to the Saline (−90.21 ± 3.28; *p* = 0.0003), Inosine (−79.49 ± 2.60; *p* = 0.0016), and Saline + TT (−83.32 ± 1.73; *p* = 0.0185) groups, indicating a synergistic benefit of the combined treatment. These findings suggest that TT, especially when combined with inosine, significantly enhances gait recovery following PNI.

Cutaneous pain sensitivity was evaluated using the pinprick test (*n* = 6) (Figure 2C). From the sixth week, the Inosine + TT group (3.16 ± 0.16) showed significantly greater responses to nociceptive stimuli compared to the Saline gro++up (1.83 ± 0.30; *p* = 0.0227). By the seventh week, both treadmill‐trained groups—Saline + TT (3.50 ± 0.22) and Inosine + TT (3.50 ± 0.22)—achieved significantly higher scores than the Saline group (2.50 ± 0.22; *p* = 0.0424). At the conclusion of the survival period, all three experimental groups—Inosine (4.33 ± 0.21), Saline + TT (4.00 ± 0.25), and Inosine + TT (4.66 ± 0.21)—demonstrated markedly improved responses relative to the Saline group (*p* = 0.0013, *p* = 0.0198, and *p* = 0.0003, respectively). These results support that both TT and inosine promote enhanced recovery of cutaneous sensory function, with the combined approach producing the most robust effect.

Tactile sensitivity was assessed using an electronic Von Frey analgesiometer (*n* = 6) (Figure 2D). As early as the second week, the Saline + TT group (1.64 ± 0.12) responded to significantly lower stimulus intensities than the Inosine + TT (2.09 ± 0.08; *p* = 0.0224), Inosine (2.45 ± 0.10; *p* = 0.0008), and Saline (2.25 ± 0.11; *p* = 0.0119) groups, indicating increased mechanical sensitivity. In the third week, the Inosine + TT group (1.49 ± 0.10) exhibited greater tactile sensitivity compared to both the Saline (2.08 ± 0.11; *p* = 0.0178) and Inosine (2.01 ± 0.10; *p* = 0.0290) groups. By the fourth week, both Inosine + TT (1.27 ± 0.06) and Saline + TT (1.23 ± 0.10) responded to significantly lower pressures than the Inosine (1.95 ± 0.09) and Saline (1.95 ± 0.10) groups (*p* < 0.005 for both comparisons). At the end of the observation period, all treated groups—Inosine (0.48 ± 0.10), Saline + TT (0.61 ± 0.07), and Inosine + TT (0.58 ± 0.08)—showed enhanced sensitivity to lower‐intensity stimuli when compared to the Saline group (0.91 ± 0.05; *p* < 0.05). These findings demonstrate that TT, particularly in combination with inosine, accelerates and enhances the recovery of tactile sensory function following nerve injury.

### 3.2 Combination of Treadmill Training and Inosine Treatment Improves Nerve Regeneration After Sciatic Nerve Transection and Repair

**FIGURE 3:**
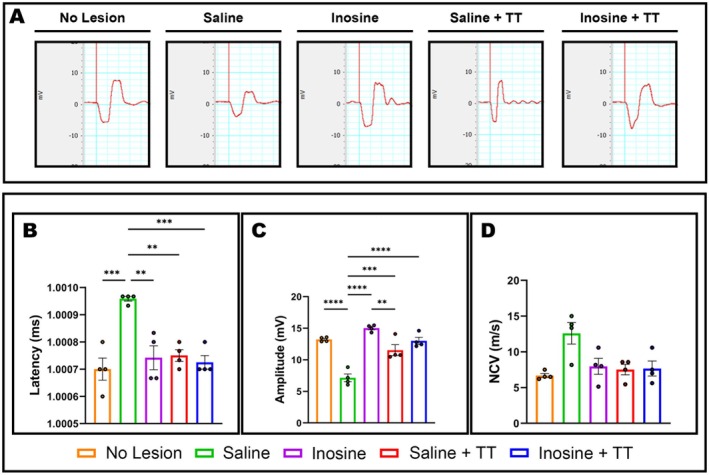
Electrophysiological analysis at 8 weeks post‐injury. (A) Compound muscle action potential (CMAP) traces for the uninjured (orange), saline (green), inosine (purple), saline + TT (red) and inosine + TT (blue) groups. Graphs showing (B) CMAP latency, (C) amplitude and (D) nerve conduction velocity (m/s) across the five groups. For electrophysiology: *N* = 4. Values represent mean ± SEM (**p < 0.005, ***p < 0.0005 and ****p < 0.0001).

To evaluate axonal regeneration through compound muscle action potential (CMAP) recordings after sciatic nerve transection and repair, electroneuromyography was performed (*n* = 4) (Figure 3). The Inosine + TT (1.0007 ± 0.000025), Saline + TT (1.0007 ± 0.000021), and Inosine (1.0007 ± 0.000043) groups displayed no significant differences in latency compared to the uninjured group (1.0007 ± 0.000040). In contrast, the Saline group exhibited a significantly prolonged latency (*p* < 0.005) (Figure 3B). Regarding CMAP amplitude, the Inosine + TT (13.00 ± 0.55; *p* < 0.0001), Saline + TT (11.53 ± 0.85; *p* = 0.0004), and Inosine (15.01 ± 0.74; *p* < 0.0001) groups demonstrated significantly greater amplitudes than the Saline group (7.15 ± 0.60), which in turn showed reduced amplitude when compared to the uninjured group (13.22 ± 0.17; *p* < 0.0001) (Figure 3C). No significant differences in nerve conduction velocity were observed among the experimental groups (Figure 3D). These findings indicate that inosine treatment, especially when combined with TT, enhances axonal regeneration by improving CMAP amplitude and restoring latency values to levels comparable with uninjured animals.

**FIGURE 4:**
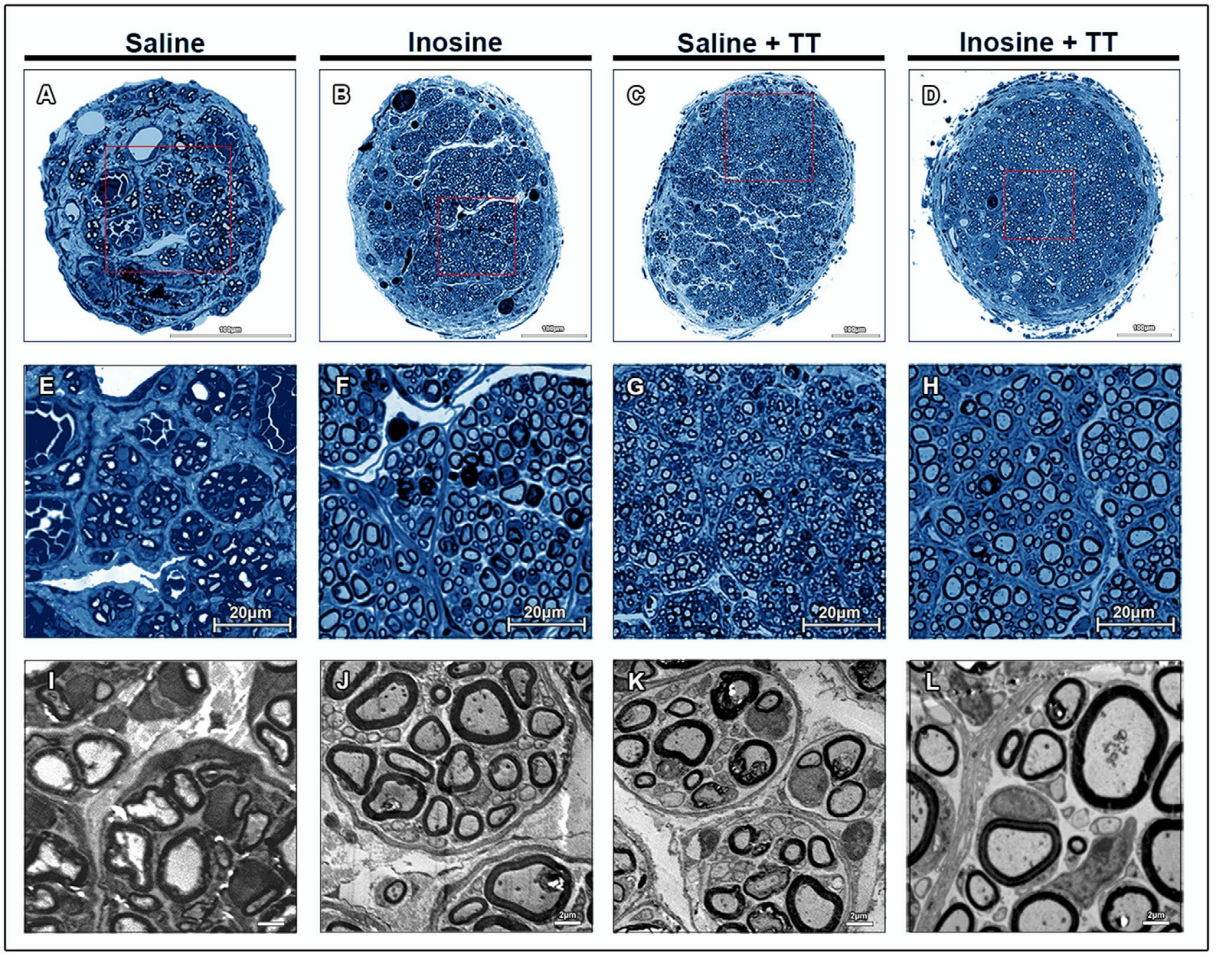
Morphology of regenerating nerves. (A–H) Semi‐thin sections of segment B of the sciatic nerves from animals in the four groups. The nerves from groups treated with inosine, treadmill training, and the combined treatment (B–D) show a larger total area and a higher number of myelinated fibers compared to the saline group (A). Scale bar = 100 μm. At higher magnification (E–H), the treated groups (F–H) appear to have a more organized environment compared to the saline group (E). Scale bar = 20 μm. (I–L) Ultra‐thin sections of segment B of the sciatic nerves from animals in the four groups. The groups treated with inosine, treadmill training, and the combined treatment (J–L) appear to have more myelinated fibers, whereas the saline group (A) appears to have more fibers in the process of myelination. Scale bar = 2 μm.

**FIGURE 5:**
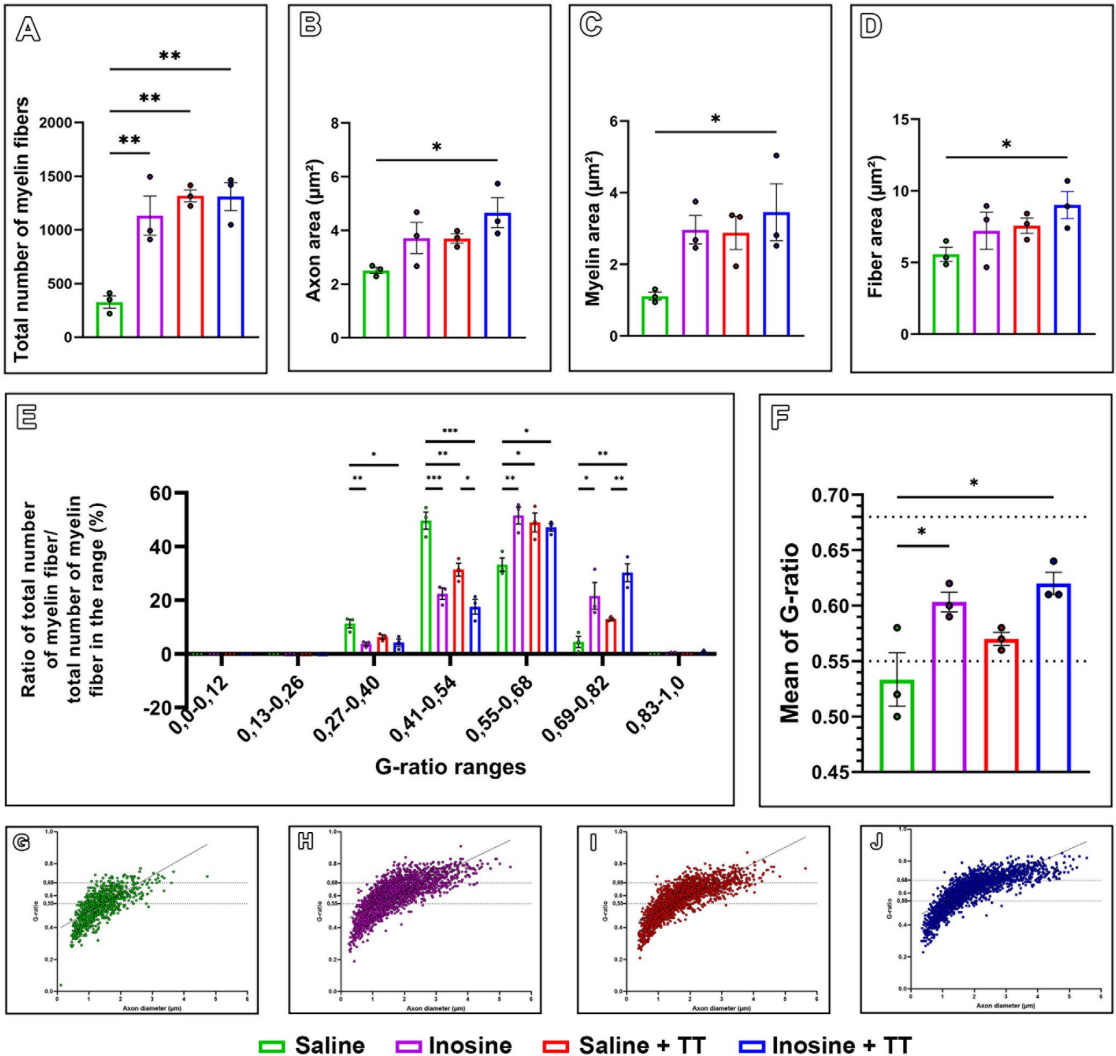
Morphometry of regenerating nerves. (A) Number of myelinated fibers. (B) Axon area (μm^2^). (C) Myelin area (μm^2^). (D) Myelinated fiber area (μm^2^). (E) G‐ratio distribution of segment B of the regenerating sciatic nerve. (F) Mean G‐ratio of segment B of the regenerating sciatic nerve. (G–J) Relationship between G‐ratios and axon diameters (μm) in the saline (H), inosine (I), saline + TT (J), and inosine + TT (K) groups. For morphometric analyses: *N* = 3. Data presented as mean ± SEM. (*p < 0.05, **p < 0.005, ***p < 0.0005).

After 8 weeks, morphometric analysis of segment B revealed improved tissue organization and a greater number of myelinated fibers in the Inosine + TT, Saline + TT, and Inosine groups compared to the Saline group (328.00 ± 56.71; *p* < 0.05) (Figures 4 and 5). The Inosine + TT group also exhibited significantly larger axonal, myelin, and total fiber areas relative to the Saline group (*p* < 0.05). Additionally, higher G‐ratios within the optimal physiological range (0.55–0.68) were observed in the Inosine + TT, Saline + TT, and Inosine groups, with statistically significant differences also detected outside this range (*p* < 0.05). These findings suggest that inosine treatment, particularly when combined with TT, promotes more effective morphological nerve regeneration, with improved myelination and structural maturation of regenerated fibers.

**FIGURE 6:**
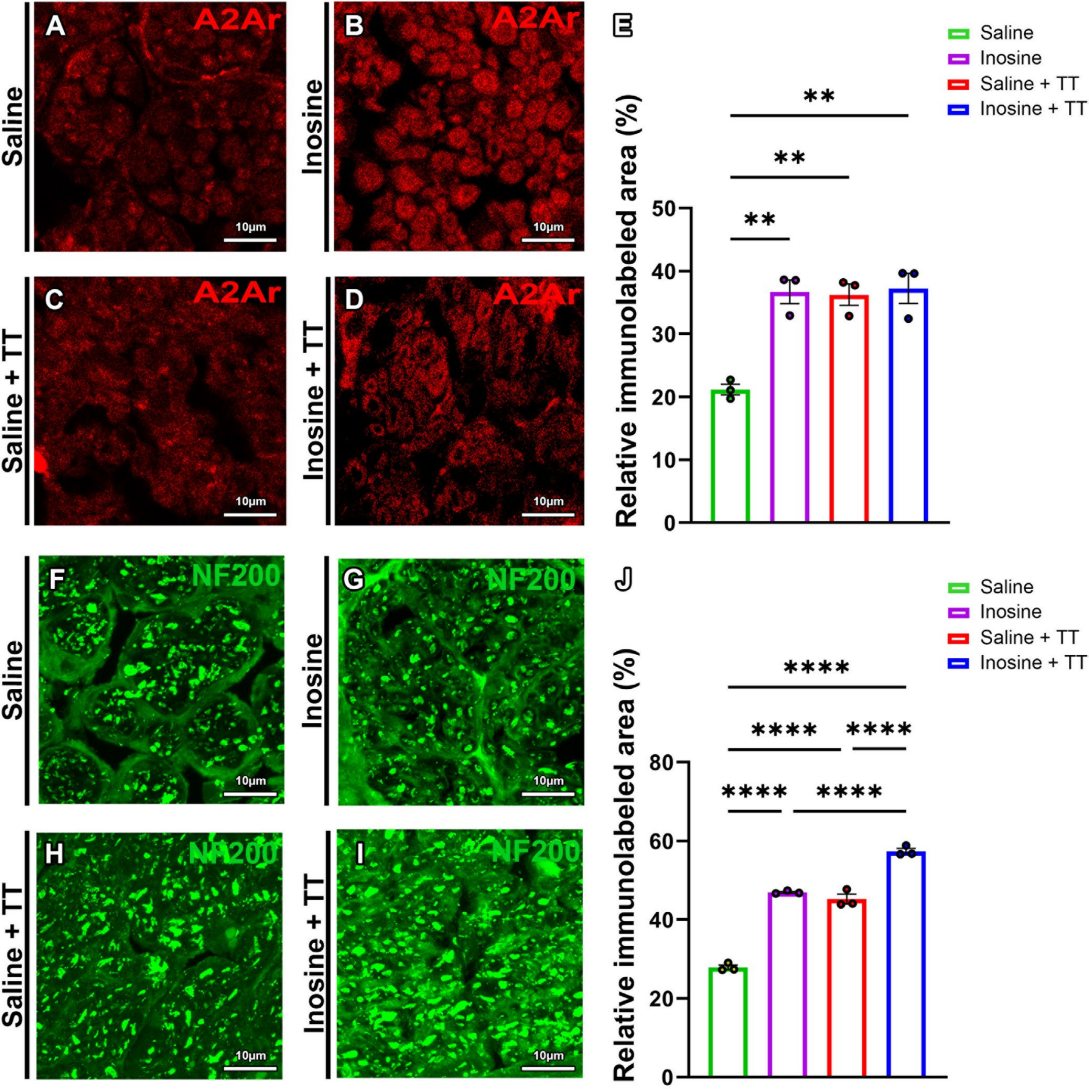
Analysis of relative immunostained areas for A2Ar and NF200. For A2Ar (A–D) note the staining intensity in the saline group (A) and increased intensity in the inosine (B), saline + TT (C), and inosine + TT (D) groups. Scale bar: 10 μm. (E) Quantification of relative immunostained area for A2A receptor (A–D). For NF‐200 (F–I) note the staining intensity in the saline group (A) and increased intensity in the inosine (B), saline + TT (C), and inosine + TT (D) groups. Scale bar: 10 μm. (J) Quantification of relative immunostained area for NF‐200. For A2Ar and NF‐200 immunostained area analysis: N = 3. Data presented as mean ± SE. (**p < 0.005 and ****p < 0.0001).

IHC analysis for A2A receptor (A2Ar) revealed more intense staining in the Inosine, Saline + TT, and Inosine + TT groups compared to the Saline group, with statistically significant differences (*p* < 0.0001) (Figure 6). Similarly, IHC staining for neurofilament 200 (NF200) demonstrated greater immunoreactivity in the Inosine, Saline + TT, and Inosine + TT groups compared to Saline, with significant differences observed (*p* < 0.0464). These results indicate that both inosine treatment and TT, independently or in combination, enhance the expression of molecular markers associated with axonal integrity and regeneration.

### 3.3 Combination of Treadmill Training and Inosine Treatment Prevents Neuronal Death in Spinal Cord Ventral Horn and DRG

**FIGURE 7:**
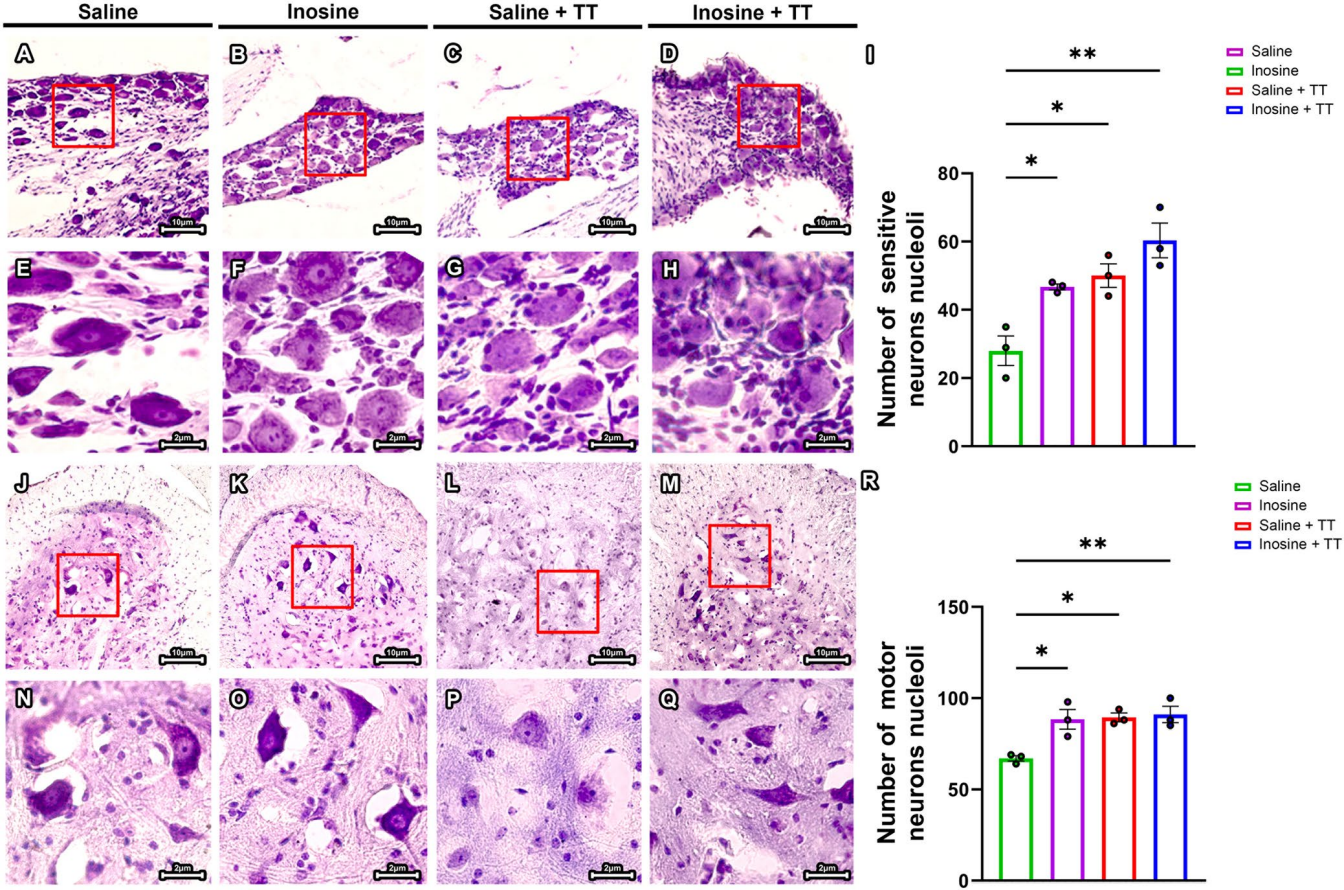
Quantification of cell bodies of motor and sensory neurons. (A–H) Transverse sections of DGR and (J–Q) Transverse sections of spinal cords, stained with 0.5% cresyl violet at 20× magnification (A–D; J–M) and 63× magnification (E–H; N–Q). (A, E, J, N) Saline; (B, F, K, O) Inosine; (C, G, L, P) Saline + TT; (D, H, M, Q) Inosine + TT. For quantitative analyses of nucleoli in sensory and motor neurons: *N* = 3. Data presented as mean ± SEM (*p < 0.05 and **p < 0.005). Scale bar: 10 μm (A–D; J–M) and 2 μm (E–H; N–Q).

To assess neuroprotective effects—specifically, neuronal survival—following sciatic nerve transection and repair, nucleoli were quantified in sensory neurons of the DRG (Figure 7A–H) and motor neurons within the anterior horn of the spinal cord (Figure 7J–Q) at 8 weeks post‐injury. All intervention groups—Inosine (46.67 ± 0.88), Saline + TT (50.00 ± 3.46), and Inosine + TT (60.33 ± 5.04)—demonstrated significantly higher nucleolar counts in DRG neurons compared to the Saline group (28.00 ± 4.35). A similar pattern was observed in spinal motor neurons, where nucleolar counts were also elevated in the Inosine (88.33 ± 5.48), Saline + TT (89.33 ± 2.40), and Inosine + TT (91.00 ± 4.58) groups relative to Saline (67.00 ± 1.52). Statistical analysis confirmed significant differences at *p* < 0.005 for Inosine + TT versus Saline, and at *p* < 0.05 for Inosine and Saline + TT versus Saline. These results indicate that inosine administration and TT, both individually and in combination, promote sensory and motor neuron survival following PNI and surgical repair, supporting their neuroprotective potential in vivo.

### 3.4 Combination of Treadmill Training and Inosine Treatment Attenuated the Loss of Gastrocnemius Muscle Weight and Hypotrophy

**FIGURE 8:**
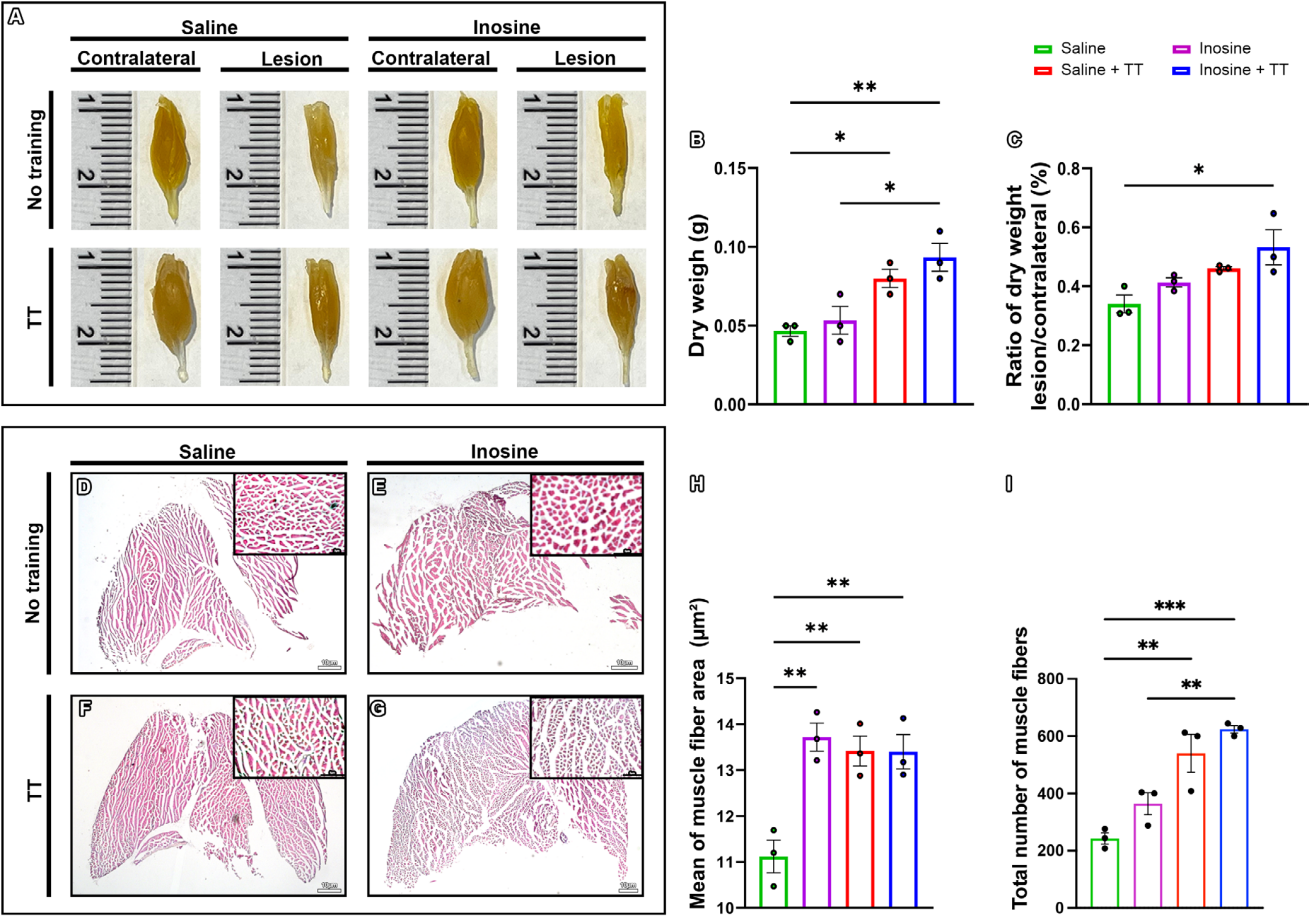
Analysis of gastrocnemius muscles. (A) Image of the right (injured) and left (uninjured control) gastrocnemius muscles from the four groups. (B) Dry weight in grams of the injured medial and lateral gastrocnemius muscles from animals in the four groups. (C) Ratio between the dry weight of injured gastrocnemius muscles and uninjured control from the four groups. For gastrocnemius muscle analyses: *N* = 3. (D–I) Morphological analysis of gastrocnemius muscles stained with HE. (A) Transverse sections of gastrocnemius muscle from groups (D) saline; (E) inosine; (F) saline + TT; and (G) inosine + TT. (H) Mean area of muscle fibers (μm^2^). (I) Number of muscle fibers. Scale bar: 10 μm. For morphological analyses of gastrocnemius muscles: *N* = 3. Data presented as mean ± SEM (*p < 0.05, **p < 0.005 and ***p < 0.0005).

At 8 weeks post‐injury, the lateral and medial gastrocnemius muscles were collected from both the injured (right) and contralateral (left) limbs across all groups. Qualitative analysis revealed visibly larger muscles bilaterally in animals subjected to the TT protocol compared to non‐trained groups (Figure 8A). Dry weight measurements of the injured limb showed significantly greater muscle mass in the Inosine + TT group (0.49 ± 0.05 g) compared to the Inosine (0.42 ± 0.04 g; *p* < 0.05) and Saline (0.33 ± 0.02 g; *p* < 0.005) groups. The Saline + TT group (0.46 ± 0.01 g) also had greater muscle weight than the Saline group (*p* < 0.05) (Figure 8B). When normalized to the contralateral limb, only the Inosine + TT group maintained a statistically significant difference (0.09 ± 0.01 vs. 0.05 ± 0.01; *p* < 0.05) (Figure 8C).

Histological evaluation of gastrocnemius cross‐sections stained with hematoxylin and eosin (H&E) (Figure 8D–G) revealed that TT groups had more abundant muscle fibers with larger cross‐sectional areas than non‐TT groups. Quantitative analysis confirmed these findings: fiber cross‐sectional areas were significantly larger in the Inosine (13.95 ± 0.32 μm^2^; *p* = 0.0004), Saline + TT (13.23 ± 0.30 μm^2^; *p* = 0.005), and Inosine + TT (13.24 ± 0.31 μm^2^; *p* = 0.0047) groups compared to Saline (11.33 ± 0.33 μm^2^) (Figure 8H). Furthermore, the number of muscle fibers was significantly higher in the Inosine + TT (624.0 ± 12.86; *p* = 0.0007) and Saline + TT (540.0 ± 66.09; *p* = 0.0033) groups relative to the Saline group (242.7 ± 19.64), with the Inosine group (364.0 ± 38.02) also showing a significant increase (*p* = 0.0075) (Figure 8I). These results indicate that TT, especially when combined with inosine treatment, effectively attenuates muscle atrophy and preserves gastrocnemius muscle morphology following sciatic nerve injury.

## 4 Discussion

The present study demonstrates that the combined application of inosine treatment and TT significantly enhances sciatic nerve regeneration and functional recovery in mice after transection injury. The surgical repair was performed using a PLA tubular conduit, a biomaterial that has shown favorable outcomes in previous studies, including increased myelinated fiber counts and improved functional and electrophysiological recovery (Cardoso et al. 2019; Chomiak and Hu 2009). These results support the hypothesis that combining molecular approaches with activity‐dependent strategies can act synergistically to optimize peripheral nerve repair.

Inosine has been extensively studied for its neuroprotective effects, acting via adenosine A2A receptor activation, promoting GAP‐43 expression, and facilitating axonal sprouting (Benowitz et al. 1999; Irwin et al. 2006; Cardoso et al. 2021). In our model, inosine alone yielded moderate improvements in functional scores and histological organization compared to the saline control, consistent with previous reports in both spinal cord and PNIs (Cardoso et al. 2019, 2024).

TT, widely recognized for its benefits in motor and sensory recovery (Gordon 2024; Bilchak et al. 2021), exerts its effects by enhancing neural plasticity and neurotrophin expression (Sabatier et al. 2008; Gordon 2020; Sun et al. 2019). In our protocol, low‐intensity TT alone resulted in functional gains over time, particularly in sensory thresholds, aligning with prior work indicating that physical activity promotes functional reorganization and remyelination (Jaiswal et al. 2020).

Both TT and inosine independently enhanced and accelerated functional recovery. Their combination produced superior outcomes, suggesting additive or synergistic effects. TT may exert its benefits through activity‐dependent mechanisms, such as the upregulation of neurotrophic factors (e.g., BDNF, NGF) and recruitment of motor neurons, which are essential for motor circuit reorganization and synaptic recovery (Jaiswal et al. 2020; de Moraes et al. 2018; Minegishi et al. 2022). Inosine, a purine nucleoside with neuroprotective and neuromodulatory properties, has shown positive effects in models of mild nerve injury (Benowitz et al. 1999; Cardoso et al. 2019, 2024; Kim et al. 2013; Paterniti et al. 2011), likely via A2A receptor activation and increased expression of neurotrophic factors (Jaiswal et al. 2020).

Motor function recovery is a primary clinical goal following PNI. The SFI revealed that the inosine + TT group significantly outperformed the saline and inosine groups, while the saline + TT group also showed improved performance over saline alone. These findings support the hypothesis that combining rehabilitation and pharmacological therapies enhances motor recovery. TT likely facilitates reconnection between motor neurons and their afferents, mitigating synaptic withdrawal and spinal circuitry reorganization (Minegishi et al. 2022).

Somatosensory recovery was also enhanced by both interventions. Eight weeks post‐injury, the inosine + TT, saline + TT, and inosine groups exhibited improved responses in the pinprick test compared to saline. TT‐treated animals responded to pressure stimuli earlier, indicating more rapid cutaneous nociceptive recovery. These results align with prior findings (Goulart and Martinez 2015; Fletcher et al. 2024), suggesting that combined interventions can facilitate sensory restoration as well.

Peripheral nerve functional recovery depends on both axonal regeneration and remyelination (Akram et al. 2022). Electrophysiological assessment via electromyography—the clinical gold standard—demonstrated that TT + inosine‐treated animals exhibited CMAP amplitudes and latencies comparable to uninjured controls. These findings are consistent with enhanced muscle contraction and improved nerve conduction (Gordon 2020), underscoring their significance in nerve injury recovery.

Histologically, the inosine + TT group displayed superior nerve organization, with larger axon, fiber, and myelin areas relative to saline. An optimal *G*‐ratio (0.55–0.68) is essential for effective impulse conduction (Chomiak and Hu 2009); this was observed in the inosine and inosine + TT groups, indicating more functional regeneration. These benefits may stem from inosine's ability to activate A2A receptors and upregulate GAP‐43, a key protein in axonal growth (Irwin et al. 2006; Ribeiro et al. 2016), and from TT's capacity to modulate receptor density and support motor plasticity (Bauer et al. 2020).

Both interventions may promote a pro‐regenerative environment by modulating neurotrophic factors systemically (Tari et al. 2019). Immunohistochemical analysis revealed increased A2A receptor expression in treated groups, particularly in Schwann cells, which are essential for nerve repair (Darabid et al. 2018). Elevated A2A receptor expression is associated with reduced inflammation, via decreased macrophage infiltration, and improved neuronal survival (Rajasundaram 2018; Awad et al. 2006; Pedata et al. 2014).

Immunostaining for NF200 confirmed more organized tissue architecture in treated animals. As axons mature, they incorporate high‐molecular‐weight neurofilament subunits that stabilize the cytoskeleton and are critical for myelinated fiber integrity (Perrot et al. 2008; Yuan et al. 2017; Goulart et al. 2016). This structural maturation supports the morphometric findings of larger axonal and myelin areas in TT‐ and/or inosine‐treated animals.

Effective axonal regeneration also relies on retrograde signaling from the lesion to the soma and on active somatic responses, including material synthesis and transport (Albus et al. 2013; Rishal and Fainzilber 2014; Zhao et al. 2024). A greater number of nucleoli in DRG and spinal motor neurons in the treated groups suggests enhanced neuronal activity and a possible neuroprotective effect.

Finally, muscle preservation is essential for functional recovery. TT‐treated animals exhibited greater ipsilateral gastrocnemius muscle mass, with the inosine + TT group maintaining this effect even after normalization. Histological analysis revealed larger muscle fibers in all treated groups, consistent with improved innervation. Reinnervation supports the recovery of muscle fiber size and nerve caliber, which are determinants of contractile function (Gordon 2020; Minegishi et al. 2022; Gordon and de Zepetnek 2016; Kong et al. 2022).

In summary, the most striking results emerged from the group receiving both inosine and TT. These animals exhibited accelerated recovery in all functional tests, improved CMAP amplitude and latency, and greater preservation of muscle and nerve morphology. These findings suggest a complementary mechanism where inosine enhances intrinsic regenerative signaling, while TT provides extrinsic stimulation to guide functional reconnection. This supports the idea that regeneration is maximized when intrinsic neuronal growth potential and target reinnervation are simultaneously optimized.

Electrophysiological analysis further supports this interpretation. The combination group demonstrated earlier reappearance and improved amplitude of CMAPs compared to all other groups, indicating faster and more effective axonal reconnection. Latency values remained longer than in naïve animals, which is expected due to incomplete remyelination but were significantly reduced compared to control and monotherapy groups.

In conclusion, the combination of inosine and TT resulted in additive benefits to nerve regeneration, surpassing either treatment alone. These results emphasize the value of multimodal therapeutic strategies that target both molecular and activity‐dependent mechanisms in peripheral nerve repair. Further studies are warranted to explore dosing regimens, long‐term outcomes, and potential clinical translation.

This study has some limitations. First, cell quantification was based on nucleolar counts, a practical and reproducible method previously employed in our group's earlier studies (Goulart and Martinez 2015; Cardoso et al. 2024). While effective for estimating neuronal survival, this approach does not provide the stereological precision of unbiased counting methods and may introduce estimation bias. Furthermore, our experimental design focused on short‐term recovery (up to 8 weeks). Although this timeframe is sufficient to assess early functional and regenerative changes, it does not capture long‐term outcomes such as sustained reinnervation, synaptic stability, or chronic remodeling.

## Author Contributions

**Tiago Batos Taboada:** conceptualization, data curation, formal analysis, investigation, methodology, visualization, writing – original draft, writing – review and editing. **Luiza dos Santos Heringer:** investigation, methodology, writing – review and editing. **Camila Linhares Fernandes de Oliveira:** writing – review and editing. **Gabriel Valladares da Rosa:** methodology, writing – review and editing. **Fernanda Marques Pestana:** methodology, writing – review and editing. **Ricardo Cardoso:** methodology, writing – review and editing. **Roberta Ramos Cavalcanti:** writing – review and editing. **Fellipe Soares dos Santos Cardoso:** data curation, investigation, methodology, writing – review and editing. **Bruna dos Santos Ramalho:** investigation, methodology, writing – review and editing. **Ana Maria Blanco Martinez:** conceptualization, data curation, formal analysis, funding acquisition, visualization, writing – original draft, writing – review and editing. **Fernanda Martins de Almeida:** conceptualization, data curation, formal analysis, funding acquisition, project administration, supervision, validation, visualization, writing – original draft, writing – review and editing.

## Conflicts of Interest

The authors declare no conflicts of interest.

## Declaration of Transparency

The authors, reviewers and editors affirm that in accordance to the policies set by the Journal of Neuroscience Research, this manuscript presents an accurate and transparent account of the study being reported and that all critical details describing the methods and results are present.

## Supporting information


**Data S1:** jnr70080‐sup‐0001‐Supinfo.docx.

## Data Availability

The data that support the findings of this study are available from the corresponding author upon reasonable request.
